# Safety and Efficacy of Novel RNA Interference Therapeutic Agent Zilebesiran in People With Hypertension: A Systematic Review and Meta-Analysis

**DOI:** 10.7759/cureus.77607

**Published:** 2025-01-18

**Authors:** Vanshika Singh, Jay Tewari, Khalid A Qidwai, Shailendra K Singh, Ajoy Tewari, Vineeta Tewari, Anuj Maheshwari

**Affiliations:** 1 Internal Medicine, King George's Medical University, Lucknow, IND; 2 Internal Medicine, King George’s Medical University, Lucknow, IND; 3 Endocrinology, Endocrine Clinic, Varanasi, IND; 4 Diabetes and Endocrinology, Jai Clinic and Diabetes Care Center, Lucknow, IND; 5 Anatomy, Era's Lucknow Medical College and Hospital, Era University, Lucknow, IND; 6 Medicine, Hind Institute of Medical Sciences, Lucknow, IND; 7 Medicine, Shri Hari Kamal Diabetes and Heart Research Clinic, Lucknow, IND

**Keywords:** blood pressure, hypertension, raas, rna interference, systematic review and meta-analysis, zilebesiran

## Abstract

Hypertension is a major risk factor for cardiovascular and renal diseases. Despite the availability of a wide variety of anti-hypertensive agents, a large number of patients on anti-hypertensive therapy have uncontrolled hypertension. Several factors, such as incorrect dosage and treatment non-adherence due to side effects, polypharmacy, and complex dosing regimens, can cause inadequate blood pressure regulation. Under phase 1 and 2 trials, Zilebesiran is a subcutaneously administered novel RNA interference therapeutic agent that reduces blood pressure by targeting angiotensinogen (AGT) synthesis. We conducted this systematic review and meta-analysis (SRMA) to evaluate the safety and efficacy of Zilebesiran in reducing blood pressure. Following the Preferred Reporting Items for Systematic Reviews and Meta-Analyses (PRISMA) 2020 guidelines, a literature search was carried out across Pubmed, Embase, and Cochrane Central databases. Our study included two randomized controlled trials (RCTs) involving adult participants receiving Zilebesiran or placebo. The primary outcomes assessed were changes in mean 24-hour ambulatory systolic and diastolic blood pressure and the incidence of adverse events at 12 weeks. Secondary outcomes included changes in serum creatinine and estimated glomerular filtration rate. Zilebesiran administration resulted in a significant reduction in 24-hour systolic blood pressure (-15.12 mmHg, 95% CI: -17.21 to -13.03) and diastolic blood pressure (-7.34 mmHg, 95% CI: -8.70 to -5.98) at 12 weeks compared to placebo with I² = 0%, indicating consistent results across trials. A statistically significant increase in total adverse events was noted (risk ratio: 1.15, 95% CI: 1.01 to 1.30), while serious adverse events did not differ significantly. The commonly encountered adverse effects included pain and erythema at the injection site and headache. Renal safety was supported by consistent serum creatinine and eGFR levels across the included studies. Zilebesiran demonstrates substantial efficacy in blood pressure reduction and exhibits a favorable safety profile, presenting a potential alternative for patients, particularly those struggling with adherence to daily oral medications. The inclusion of only two RCTS resulted in a small sample size, predominantly composed of white participants. This limits the generalizability of the results and highlights the need for further trials with a larger and more diverse study population. Further long-term investigations are also needed to establish the drug's impact on body weight, blood glucose levels, lipid profile, cardiovascular and hepatic health, and its efficacy when used alongside conventional antihypertensive medications.

## Introduction and background

Hypertension, often referred to as the “silent killer,” is a leading risk factor for cardiovascular and renal diseases including cerebrovascular disease, ischemic heart disease, and chronic kidney disease [1,2]. According to the World Health Organization (WHO) fact sheet (2023), globally an estimated 1.28 billion people between the ages of 30 and 79 have hypertension with around two-thirds living in low- and middle-income countries. The risk of developing hypertension increases with advancing age, high salt consumption, obesity, sedentary lifestyle, and excessive alcohol consumption [3]. Current treatment for hypertension mainly involves lifestyle modifications and the use of oral antihypertensive agents such as angiotensin-converting enzyme inhibitors (ACEis), angiotensin II receptor blockers (ARBs), calcium channel blockers (CCBs), diuretics, beta-blockers, etc. [4]. Despite the availability of a wide variety of antihypertensive agents, only one out of every five (about 21%) individuals undergoing treatment have their blood pressure under control [3]. Several factors, such as incorrect dosage and treatment non-adherence can cause inadequate blood pressure regulation. Factors for non-adherence include medication side effects such as tiredness, dizziness, male sexual dysfunction, polypharmacy, complex dosing regimens, and poor patient-provider relationship. This emphasizes the necessity of investigating novel medications that do not require frequent dosing and can be used as adjuvant drugs alongside oral antihypertensives.

Reducing blood pressure by silencing liver-derived angiotensinogen (AGT) is a novel approach to renin-angiotensin-aldosterone system (RAAS) blockade [5]. A subcutaneously delivered investigational RNA interference medication called Zilebesiran binds to the hepatic asialoglycoprotein receptor, lowering hepatic AGT messenger RNA (mRNA) levels. This then leads to a reduction in circulating AGT levels and decreases blood pressure through modulation of the RAAS [6]. This approach poses several potential advantages over the existing therapies. Firstly, modulating RAAS at the hepatic level may exhibit a better renal safety profile. Secondly, direct inhibition of AGT may reduce escape mechanisms and provide a more thorough inhibition as compared to angiotensin-converting enzyme inhibition and angiotensin receptor blockade [5]. Lastly, Zilebesiran may be administered subcutaneously every three or six months to help address the problem of oral antihypertensive non-adherence.

This systematic review and meta-analysis (SRMA) aims to assess the efficacy and safety of Zilebesiran in lowering blood pressure across clinical trials. By integrating data from multiple studies, we seek to evaluate its potential as an effective therapeutic option for patients with hypertension.

## Review

Methods

The Preferred Reporting Items for Systematic Reviews and Meta-Analyses (PRISMA) criteria were adhered to, and the Cochrane Handbook for Systematic Reviews of Interventions principles were followed in this meta-analysis [7]. The study protocol is registered with PROSPERO under registration number CRD42024604190.

Study Selection Criteria

Studies with adult patients were considered. Studies that met the eligibility criteria had to have a minimum of two treatment groups: one that received Zilebesiran, and the other that received a placebo. The following Population, Intervention, Comparison, Outcomes, and Study (PICOS) criteria were used to identify and select studies for this meta-analysis.

The study population (P) was classified as adult people (18 to 75 years of age) with hypertension. The administration of Zilebesiran was the Intervention (I). Control (C) was classified as people receiving a placebo. Outcomes (O) were measured as Changes from baseline in ambulatory systolic blood pressure (SBP), ambulatory diastolic blood pressure (DBP), serum creatinine, estimated glomerular filtration rate (eGFR), and safety profile (serious and total adverse events (TAEs)). Only randomized controlled trials (RCTs) were included (Study design).

Search Strategy

An electronic search was undertaken in Pubmed, Embase, and Cochrane Central databases using the search terms: (Zilebesiran) or (ALN-AGT01) or (AD-85481) or (ALN-85481). The comprehensive search strategy is shown in Table 3 in the appendix.

Data Extraction and Study Selection

Two authors (VS and JT) extracted the data independently using standard data extraction forms. Along with data on the study's outcome, characteristics like author names, study design, year of publication, country of origin, experimental and comparator doses, sample size, and study duration were extracted. Any disagreements were resolved by the third author (KAQ). Data were estimated from the graphical representations provided in the original publications, where the missing data could not be obtained by contacting the authors of the publications.

Risk of Bias Assessment

Three authors (VS, JT, and KAQ) independently assessed the risk of bias using the Revised Risk of Bias Tool for Randomized Trials (RoB2) [8]. Under the following domains, the RCTs were rated as low risk, having some concerns, or high risk: bias resulting from the randomization process; bias resulting from deviations from intended interventions; bias resulting from missing outcome data; bias in the measurement of the outcome; and bias in the selection of the reported result. Discrepancies were resolved by the fourth author (SKS).

Measures of Treatment Effect

Mean differences (MDs) were used to report results for continuous variables. Conventional units were used for analysis, and SI units were converted when necessary. Risk ratios (RRs) with 95% confidence intervals (CI) were used to depict adverse events. RStudio (Posit PBC, Boston, USA) was used to compare the MDs of primary and secondary outcomes between the Zilebesiran and control groups.

Heterogeneity Assessment

Heterogeneity was also evaluated using the I^2^ test and 𝜏^2^ test. A p-value of less than 0.5 signifies the presence of significant heterogeneity. The degree and direction of treatment effects as well as the quality of the evidence supporting heterogeneity determined the significance of I^2^ values. 

Data Synthesis

A random- or fixed-effects model was used to pool the data to analyze the outcomes, which were reported as 95% CI. Forest plots were created using the meta package in Rstudio, with the left side of the graph supporting Zilebesiran and the right side supporting control. Statistical significance was attained when the p-value was less than 0.05. For pooling the MDs inverse-variance method was used and for pooling the RRs Mantel-Haenszel method was used [9].

Results

Study Selection and Characteristics

**Figure 1:**
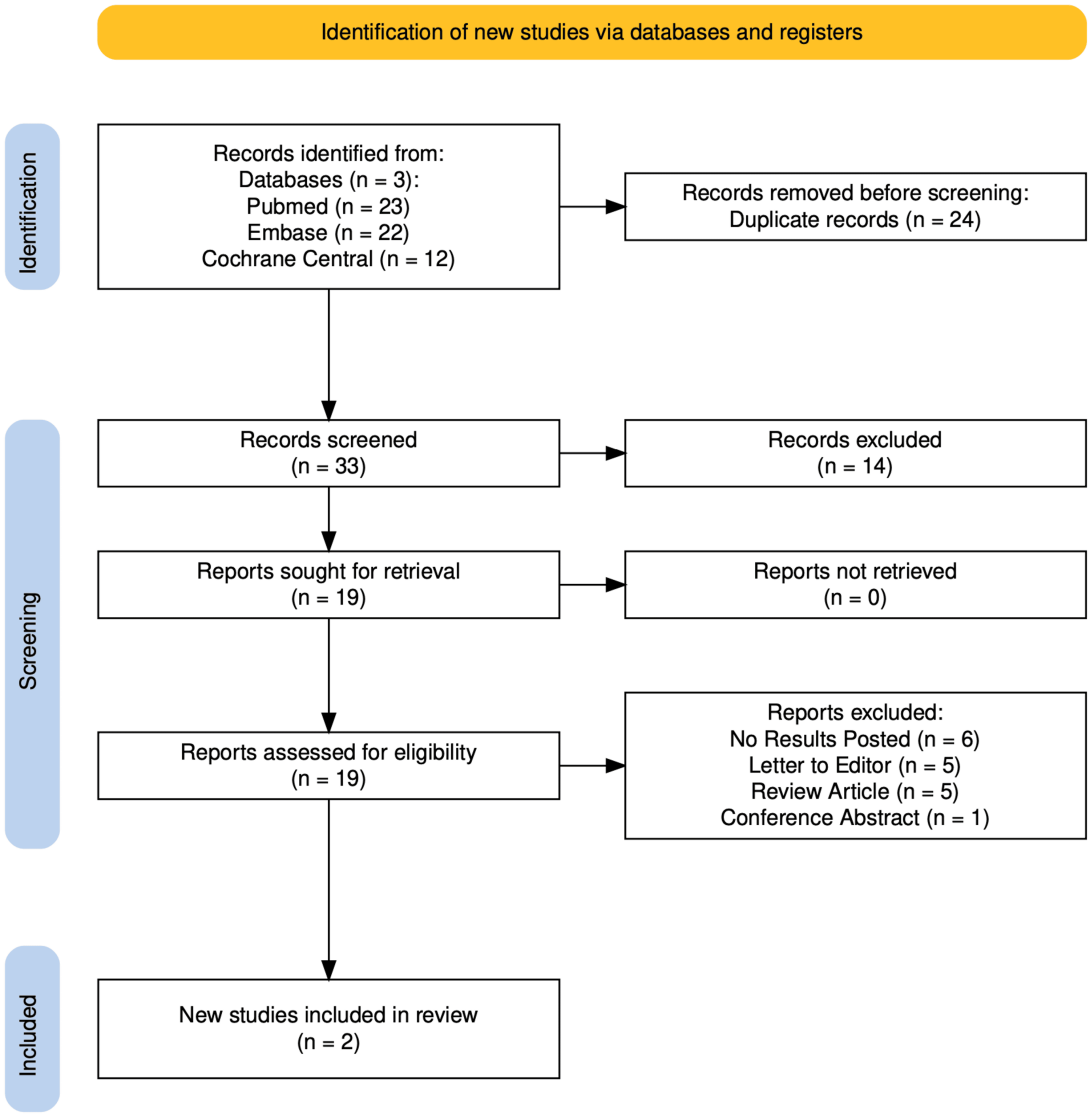
PRISMA study flow diagram

A thorough literature search was undertaken on three databases, viz., Pubmed, Cochrane Central, and Embase. The search was conducted from inception till October 22, 2024. A total of 57 articles were identified after the initial search. Deduplication yielded the deletion of 24 articles. After the final screening and assessment, two articles/trials were included in this SRMA. The PRISMA study flow diagram has been shown in Figure 1.

The characteristics of the included trials and baseline demographic information of the included people have been shown in Tables 1 and 2, respectively. The included trials used automated devices for the measurement of 24-hour SBP and DBP. Due to the different dosages in the treatment arms of the trials, we categorized the data into two subgroups (150-300 mg and 400-800 mg) to evaluate the variations in mean 24-hour SBP and DBP with different dose ranges.

**Table 1:** Characteristics of the included trials SC: Subcutaneous; OD: Once Daily. Reference: [6,10]

Author	Year	Phase of trial	Country of trial	Treatment arms	Sample size	Follow-up duration
Desai et al. [6]	2023	1	United Kingdom	Part A		
				10 mg SC	8	24 weeks
				25 mg SC	8	
				50 mg SC	8	
				100 mg SC	8	
				200 mg SC	8	
				400 mg SC	8	
				800 mg SC	8	
				Placebo	28	
				Part B		
				800 mg SC	8	12 weeks
				Placebo	4	
				Part C		
				800 mg SC	6	12 weeks
				800 mg SC + Irbesartan 300 mg OD for 2 weeks	10	
Bakris et al. [10]	2024	2	Canada, Ukraine, the United Kingdom, and the United States of America	150 mg every 6m SC	78	24 weeks
				300 mg every 6m SC	73	
				300 mg every 3m SC	75	
				600 mg every 6m SC	76	
				Placebo	75	

**Table 2:** Population baseline characteristics for the included trials BMI: Body-Mass Index; SD: Standard Deviation; NR: Not Reported Reference: [6,10]

Author	Sample age	% Males	BMI (SD) (kg/m^2^)		24-hour ambulatory SBP, mean (SD) (mmHg)	24-hour ambulatory DBP, mean (SD) (mmHg)	Type 2 diabetes mellitus (%)	Race											
								White			Black			Asian			Other		
Desai et al. (Part A) [6]	18 to 65 years	60.71	NR		140.3 (9.0)	NR	0	56			22			3			3		
																				Bakris et al. [10]	18 to 75 years	66.67	Placebo	29.3 (3.1)	142 (8)	82 (8)	17.25	259			93			23			2		
Zilebesiran	28.6 (3.0)																																						

Risk of Bias Assessment

**Figure 2:**
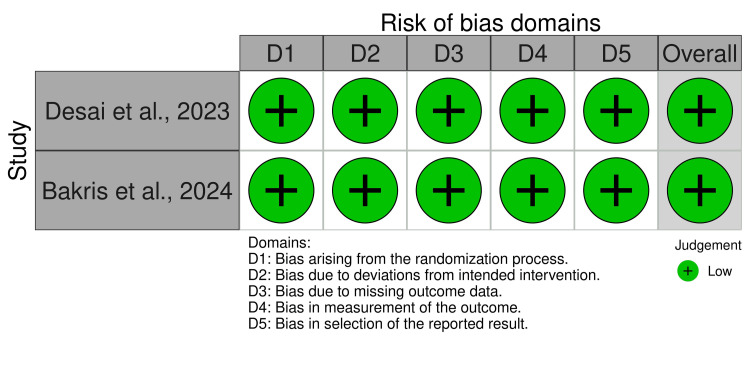
Risk of bias traffic light plot

**Figure 3:**
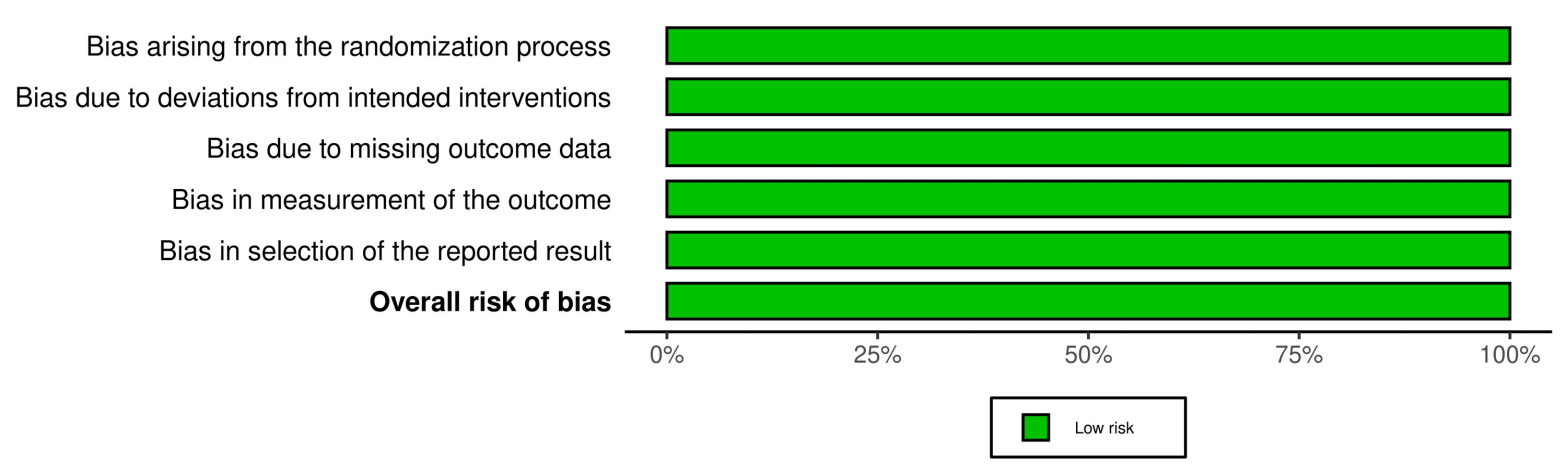
Risk of bias summary plot

The risk of bias assessment was done using the RoB2 (Cochrane, Canada) [8]. The ROB table (Figure 2) and summary plot (Figure 3) were generated using the Robvis tool (Cochrane) [11]. Across all of the domains, both trials exhibited a low level of risk.

Change in Mean 24-Hour SBP From Baseline at 12 Weeks

Dose = 150-300 mg: In this subgroup, the SBP mean reduction after 12 weeks of Zilebesiran therapy compared to the control group was observed. The overall MD was -15.56 mmHg (95% CI: -18.55 to -12.57), a statistically significant result. No heterogeneity was observed among the studies (I^2^ = 0%).

Dose = 400-800 mg: In this subgroup, the overall MD was -14.70 mmHg (95% CI: -17.62 to -11.77), a statistically significant result. No heterogeneity was observed among the studies (I^2^ = 0%).

Overall Analysis

**Figure 4:**
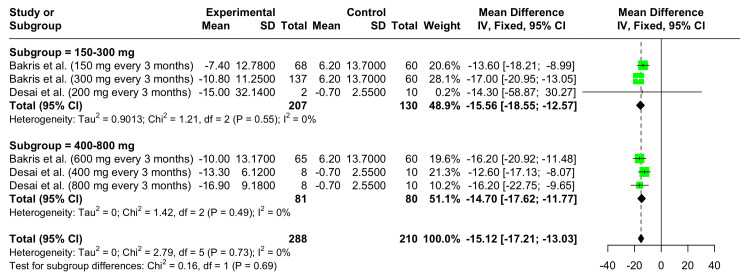
Forest plot demonstrating the efficacy of Zilebesiran versus control showing changes in the mean 24-hour SBP at 12 weeks References: [6,10]

The overall analysis (Figure 4) shows a significant reduction in mean 24-hour SBP with Zilebesiran therapy across all doses and studies, with an overall MD of -15.12 mmHg (95% CI: -17.21 to -13.03). No heterogeneity was observed across the dose groups and studies (I^2^ = 0%).

Change in Mean 24-Hour DBP From Baseline at 12 Weeks

Dose = 150-300 mg: In this subgroup, the overall MD was -8.73 mmHg (95% CI: -10.87 to -6.59), a statistically significant result. No heterogeneity was observed among the studies (I^2^ = 0%).

Dose = 400-800 mg: In this subgroup, the overall MD was -6.39 mmHg (95% CI: -8.16 to -4.63), a statistically significant result. No heterogeneity was observed among the studies (I^2^ = 0%).

Overall Analysis

**Figure 5:**
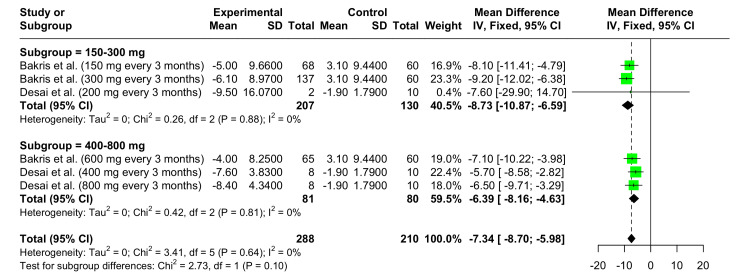
Forest plot demonstrating the efficacy of Zilebesiran versus control showing changes in the mean 24-hour DBP at 12 weeks References: [6,10]

The overall analysis (Figure 5) shows a significant reduction in mean 24-hour DBP with Zilebesiran therapy across all doses and studies, with an overall MD of -7.34 mmHg (95% CI: -8.70 to -5.98). No heterogeneity was observed across the dose groups and studies (I^2^ = 0%).

Change in Mean Serum Creatinine Level From Baseline at 12 Weeks

**Figure 6:**
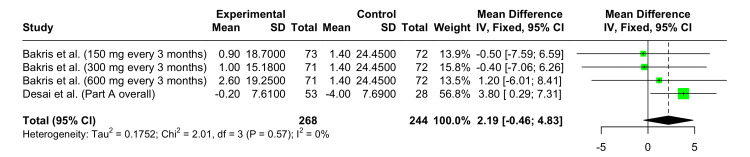
Forest plot showing the safety of Zilebesiran versus placebo demonstrating change from baseline in mean serum creatinine levels at 12 weeks References: [6,10]

In the forest plot shown in Figure 6, the pooled MD in serum creatinine change at 12 weeks is 2.19 (95% CI: -0.46 to 4.83). This result was not statistically significant. No heterogeneity was observed across the dose groups and studies (I^2^ = 0%).

Change in Mean eGFR From Baseline at 12 Weeks

**Figure 7:**
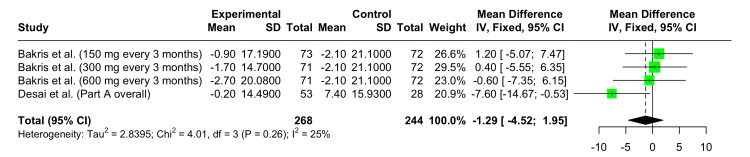
Forest plot showing the safety of Zilebesiran versus placebo demonstrating change from baseline in mean eGFR at 12 weeks References: [6,10]

In the forest plot shown in Figure 7, the pooled MD in eGFR change at 12 weeks is -1.29 (95% CI: -4.52 to 1.95). This result was not statistically significant. Some heterogeneity was observed across the dose groups and studies (I^2^ = 25%).

Total Adverse Events 

**Figure 8:**
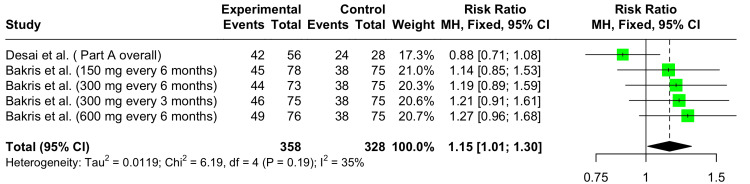
Forest plot showing the safety of Zilebesiran versus placebo showing relative risks for total adverse events at 12 weeks References: [6,10]

In the forest plot shown in Figure 8, the pooled RR for TAEs at 12 weeks across all studies is 1.15 (95% CI: 1.01 to 1.30), a statistically significant result. Some heterogeneity was observed across the dose groups and studies (I^2^ = 35%).

Serious Adverse Events

**Figure 9:**
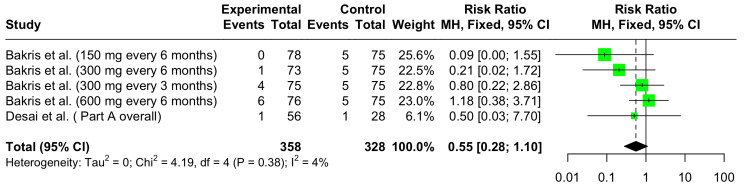
Forest plot showing the safety of Zilebesiran versus placebo showing relative risks for serious adverse events at 12 weeks References: [6,10]

In the forest plot shown in Figure 9, the pooled RR for serious adverse events (SAEs) at 12 weeks across all studies is 0.55 (95% CI: 0.28 to 1.10). This result was not statistically significant. Some heterogeneity was observed across the dose groups and studies (I^2^ = 35%).

Discussion

The development of small interfering RNA (siRNA) drugs has created new opportunities for controlling gene expression. One such investigational RNA interference therapeutic agent is Zilebesiran which exhibits anti-hypertensive properties by inhibiting hepatic AGT synthesis. In our SRMA, we evaluated the safety and efficacy of this novel antihypertensive agent.

Administration of Zilebesiran (dose > 100 mg) resulted in a decrease of more than 90% in serum AGT in studies conducted by Desai et al. and Bakris et al. A single dose (150-800 mg, administered subcutaneously) of Zilebesiran, in the experimental group, led to a significant reduction from baseline in mean 24-hour SBP (-15.12 mmHg (95% CI: -17.21 to -13.03)) and DBP (-7.34 mmHg (95% CI: -8.70 to -5.98)) at 12 weeks. Greater reduction in 24-hour SBP and DBP (-15.56 mmHg (95% CI: -18.55 to -12.57) and -8.73 mmHg (95% CI: -10.87 to -6.59) respectively) was noted in the 150-300 mg subgroup as compared to the 400-800 mg subgroup, suggesting that a lower dosage of 150-300 mg is sufficient to produce the maximal therapeutic response. The analyses for changes in mean 24-hour SBP and DBP exhibited low heterogeneity, likely due to similarity in the study population, uniform follow-up periods, and the implementation of subgroup analyses. The reduction from baseline in mean SBP and DBP with Zilebesiran is comparable to other oral antihypertensive agents. In the study by Wu et al., ACE inhibitors led to a mean reduction of 12.5 mmHg and 9.5 mmHg in SBP and DBP, respectively, over a period ranging from one week to three years. SBP and DBP were noted to drop by an average of 15.5 mmHg and 11.7 mmHg, respectively, after therapy with α1-blockers. Other drugs such as CCBs, β1-blockers thiazide, diuretics loop diuretics lowered the SBP and DBP by 15.3/10.5 mmHg, 14.8/12.2 mmHg, 15.3/9.8 mmHg, and 15.8/8.2 mmHg, respectively [12]. This drug’s ability to optimally reduce BP with quarterly and biannual dosing makes it a potential candidate for being used as a first-line antihypertensive, helping ease the burden on the healthcare system.

This subcutaneous medication also has the potential to be utilized as an adjuvant with first-line medications, particularly in non-adherent individuals. Further research is required to establish its safety and efficacy when used along with other antihypertensive agents.

In our SRMA, Zilebesiran exhibited a statistically significant increase in RR (1.15 (95% CI: 1.01 to 1.30)) of TAE at 12 weeks across both studies in comparison to placebo. The commonly encountered AEs included pain and erythema at the injection site and headache. No significant increase in the RR for SAE was noted. There were no statistically significant variations in the mean serum creatinine levels and eGFR, indicating renal safety. Nevertheless, in the study conducted by Bakris et al. [10], several episodes of hypotension, hyperkalemia, acute kidney failure, and hepatic AEs were recorded. This underscores the need for further trials involving a larger and more diverse study population to more comprehensively establish the drug's safety. No deaths or unplanned hospitalizations due to heart failure or stroke, related to drug use were reported in the trials. The analyses for TAEs, SAEs, and eGFR exhibited some heterogeneity (I^2^ = 25%-35%) which likely stemmed from the absence of subgroup analyses. Being an AGT synthesis inhibitor, this medication can offer a more complete RAAS blockade and circumvent the RAAS escape phenomenon that occurs with ACE inhibitors and ARBs. However, complete RAAS blockade can be potentially harmful in situations where severe hypotension occurs and the RAAS is required, such as when a woman becomes pregnant while undergoing therapy. These incidents necessitate additional investigation to prove the drug's safety [13].

IONIS-AGT-LRx developed by Ionis Pharmaceuticals, Carlsbad, California, is another medication blocking AGT synthesis. This is an antisense-oligonucleotide (ASO) drug that targets the hepatic mRNA and consequently reduces AGT levels in the plasma. A study by Morgan ES et al. [5] reported a substantial drop of -17.0 ± 4.1 μg/mL in serum AGT levels, at day 57 in the participant group receiving 80 mg IONIS-AGT-LRx subcutaneously. However, unlike Zilebesiran, no significant reductions were noted in SBP and DBP levels following the administration of this drug [5].

RAAS pathway hyperactivity over time has been found to have a significant role in the etiology of cardiovascular diseases, such as heart failure and hypertension [14,15]. Research on these medications' cardioprotective effects should therefore be a priority.

Our SRMA is the first to evaluate data from trials conducted on Zilebesiran. The drug's safety and effectiveness have been thoroughly established by combining data from phase 1 and phase 2 trials. The research incorporated in this SRMA was characterized by excellent scientific standards and a low likelihood of bias. The heterogeneity was low indicating consistent results. A placebo was employed as the comparison in each of the included trials, enhancing the validity of the results. The results show a lot of promise, and we eagerly await the completion of further trials for more robust results. This SRMA shall serve as a foundation for future SRMAs evaluating siRNA medications for hypertension.

Only two trials have posted their findings thus far, resulting in a small sample size. Moreover, white people made up the bulk of the participants, limiting the analysis’ generalizability. Being a siRNA drug, Zilebesiran is likely to be expensive, with its initial distribution limited to high-income countries. Therefore, pharmaceutical companies must ensure its distribution across diverse regions to enable a more thorough assessment of its efficacy and generate more broadly applicable results. In the study conducted by Desai et al. [6], CIs for changes in mean 24-hour SBP and DBP had to be approximated as we were unable to obtain the data. Data about the drug's effect on weight, blood glucose levels, lipid profile, and cardiovascular health are required for further evaluation of the long-term effects of the drug. Zilebesiran is further being evaluated as an antihypertensive agent under three phase two trials namely, KARDIA 2 (NCT05103332) [16], KARDIA 3 (NCT06272487) [17], and NCT06423352 [18]. The KARDIA 1 (ClinicalTrials.gov number, NCT04936035) [19] extension phase is in progress.

## Conclusions

This SRMA highlights Zilebesiran's potential as an effective antihypertensive agent, demonstrating substantial reductions in both SBP and DBP. Furthermore, the drug also exhibits a favorable safety profile with no significant changes in serum creatinine levels and eGFR and a statistically insignificant risk of TAEs. Its quarterly or biannual delivery schedule offers a novel strategy for long-term blood pressure control by addressing a crucial adherence concern frequently connected to daily oral regimens. Zilebesiran may also be beneficial as an adjuvant therapy for people requiring combination therapy. However, additional long-term research with a larger and more diverse study population is necessary to enhance the generalizability of the results and further evaluate Zilebesiran’s safety and efficacy when used with conventional antihypertensive medications. Additionally, studies should also explore its possible cardiovascular implications and long-term impacts on renal and hepatic health.

## Appendices

**Table 3:** Search strategy Date of search = October 22, 2024

Search engine	Search criteria	Number of unique items retrieved
PubMed	“Zilebesiran” OR “ ALN-AGT01” OR “AD-85481” OR ” ALN-85481”	23
Cochrane	#1 Zilebesiran	11
	#2 ALN-AGT01	4
	#3 AD-85481	0
	#4 ALN-85481	1
	#1 OR #2 OR #3 OR #4	13
Medline	("Zilebesiran" or " ALN-AGT01" or "AD-85481" or " ALN-85481").mp. [mp=title, book title, abstract, original title, name of substance word, subject heading word, floating sub-heading word, keyword heading word, organism supplementary concept word, protocol supplementary concept word, rare disease supplementary concept word, unique identifier, synonyms, population supplementary concept word, anatomy supplementary concept word]	22

## References

[1] Forouzanfar MH, Liu P, Roth GA (2017). Global burden of hypertension and systolic blood pressure of at least 110 to 115 mm Hg, 1990-2015. JAMA.

[2] Danaei G, Ding EL, Mozaffarian D, Taylor B, Rehm J, Murray CJ, Ezzati M (2009). The preventable causes of death in the United States: comparative risk assessment of dietary, lifestyle, and metabolic risk factors. PLoS Med.

[3] (2024). Hypertension. https://www.who.int/news-room/fact-sheets/detail/hypertension.

[4] Carey RM, Moran AE, Whelton PK (2022). Treatment of hypertension: a review. JAMA.

[5] Morgan ES, Tami Y, Hu K (2021). Antisense inhibition of angiotensinogen with Ionis-AGT-l(rx): results of Phase 1 and Phase 2 studies. JACC Basic Transl Sci.

[6] Desai AS, Webb DJ, Taubel J (2023). Zilebesiran, an RNA interference therapeutic agent for hypertension. N Engl J Med.

[7] Page MJ, McKenzie JE, Bossuyt PM (2021). The PRISMA 2020 statement: an updated guideline for reporting systematic reviews. BMJ.

[8] Sterne JA, Savović J, Page MJ (2019). RoB 2: a revised tool for assessing risk of bias in randomised trials. BMJ.

[9] R Core Team (2024). A Language and Environment for Statistical Computing. https://www.R-project.org/.

[10] Bakris GL, Saxena M, Gupta A (2024). RNA interference with Zilebesiran for mild to moderate hypertension: the Kardia-1 randomized clinical trial. JAMA.

[11] McGuinness LA, Higgins JP (2021). Risk-of-bias VISualization (robvis): an R package and Shiny web app for visualizing risk-of-bias assessments. Res Synth Methods.

[12] Wu J, Kraja AT, Oberman A (2005). A summary of the effects of antihypertensive medications on measured blood pressure. Am J Hypertens.

[13] Cruz-López EO, Ye D, Wu C, Lu HS, Uijl E, Mirabito Colafella KM, Danser AH (2022). Angiotensinogen suppression: a new tool to treat cardiovascular and renal disease. Hypertension.

[14] Ferrario CM, Strawn WB (2006). Role of the renin-angiotensin-aldosterone system and proinflammatory mediators in cardiovascular disease. Am J Cardiol.

[15] Maryam Maryam, Varghese TP, B T (2024). Unraveling the complex pathophysiology of heart failure: insights into the role of renin-angiotensin-aldosterone system (RAAS) and sympathetic nervous system (SNS). Curr Probl Cardiol.

[16] (2024). A randomized, double-blind, placebo-controlled, multicenter study to evaluate the efficacy and safety of Zilebesiran used as add-on therapy in patients with hypertension not adequately controlled by a standard of care antihypertensive medication. clinicaltrials.gov.

[17] (2024). A randomized, double-blind, placebo-controlled, multicenter study to evaluate the efficacy and safety of Zilebesiran used as add-on therapy in adult patients with high cardiovascular risk and hypertension not adequately controlled by standard of care. clinicaltrials.gov.

[18] (2024). A phase 1/2, randomized, double-blind, placebo-controlled, parallel-group study to evaluate the safety, tolerability, efficacy, pharmacodynamics, and pharmacokinetics of Zilebesiran in Japanese patients with mild to moderate hypertension. clinicaltrials.gov.

[19] (2024). A randomized, double-blind, placebo-controlled, dose-ranging multicenter study to evaluate the efficacy and safety of AlN-Agt01 in patients with mild-to-moderate hypertension. clinicaltrials.gov.

